# Rubber Attachment

**Published:** 1862-03

**Authors:** 


### RUBBER ATTACHMENT.

The attachment of teeth to gold plates by rubber is a subject that has received some attention by a few in the profession; and we confess that till quite recently, our appreciation has not been as great as the merit of the work would warrant.

The method of doing the work has been described; but perhaps by none better than by Dr. Hunt, of Indianapolis, in the July number of the Register, for 1860.

Dr. Hunt's description is very good, and to it we would refer all those who feel an interest in the matter, as we do not propose here to give a full description of the method, but only to make some suggestions in regard to it, and state its advantages.

This style of work may be employed in a large proportion of cases.

By this method the plate retains its adaptation during the process of construction perfectly; it remains just as it comes from the die. If the impression is good and the casts correct, the plate will fit at the completion of the operation. This immunity from change is by no means a small matter.

Again, it has equal, if not greater strength than any other kind of work. The base of each tooth or section has a socket formed of the rubber in which to rest; so that the teeth do not depend upon the rivets only for their support, as is the case with the ordinary gold work; and with the pin-head rivets for the teeth, as now made by S. S. White, it is impossible to draw the teeth from their position without either breaking the pins or cutting away the rubber.

Then again there is elasticity of the attachment, sufficient to accommodate any ordinary jar from a fall or percussion. There is in this respect some advantage over gold or continuous gum work. The openings, joints or interstices are all closed, so that it is easily kept clean—is as good in this respect as continuous gum work, and far superior to the ordinary gold work. The unpleasant rattling sound sometimes experienced by the occlusion of gold or continuous gum dentures, is wholly avoided by the rubber attachment. "Plumpers" for restoring the contour of the face may be employed with this kind of work with the utmost facility; and these in any form, size or position.

The thickness of rubber plates is often objectionable; that objection is removed by the use of the gold plate, and it is not heavier than when made wholly of rubber, at least when equal strength is attained. It is repaired or changed with the utmost facility, with less risk and more perfectly than any other kind of work. The work can be taken down and put up again as often as may be desired, without the least injury to either the teeth or plate.

We have now only attempted to point out some of the advantages of this kind of work. In the next number we shall describe the method of constructing the work in detail, at least so far as it may be necessary. T.

